# The diagnostic accuracy of exosomes for glioma: A meta-analysis

**DOI:** 10.17305/bb.2024.11268

**Published:** 2024-10-22

**Authors:** XiangMin Zhang, YanDi Tan, XiaoYa He, Jie Huang, XiaoYing Ni, Qian Hu, JinHua Cai

**Affiliations:** 1 Department of Radiology, Children’s Hospital of Chongqing Medical University, National Clinical Research Center for Child Health and Disorders, Ministry of Education Key Laboratory of Child Development and Disorders, Chongqing, China; 2 Chongqing Engineering Research Center of Stem Cell Therapy, Chongqing, China; 3 Department of Ultrasound, The Third Affiliated Hospital of Chongqing Medical University, Chongqing, China

**Keywords:** Biomarkers, diagnosis, exosomes, meta, glioma

## Abstract

Glioma is one of the most prevalent primary intracranial tumors, and biomarker testing offers a non-invasive modality with high diagnostic efficiency. The aim of this meta-analysis is to evaluate the diagnostic effectiveness of exosomes as biomarkers for glioma. We included 16 studies on exosomes as biomarkers for gliomas. The pooled sensitivity (SEN), specificity, positive diagnostic likelihood ratio, negative diagnostic likelihood ratio, diagnostic odds ratio, and area under the curve for 25 biomarkers across these 16 studies were as follows: 82% (95% confidence interval [CI]: 0.77–0.86), 91% (95% CI: 0.86–0.94), 9.10 (95% CI: 5.64–14.68), 0.20 (95% CI: 0.16–0.25), 45.94 (95% CI: 25.40–83.09), and 0.92 (95% CI: 0.89–0.94), respectively. Meta-regression indicated that biomarker analysis, biomarker type, and sample size may be sources of heterogeneity. Subgroup analysis suggested that ultracentrifugation was a better method for extracting exosomes. microRNA and other RNA groups (small non-coding RNAs, long non-coding RNA, and circular RNA) provided higher SEN (0.88 vs 0.84 vs 0.78) compared to proteins. This study demonstrates the superior diagnostic efficacy of exosomes as biomarkers for gliomas, with high accuracy in diagnosing gliomas.

## Introduction

Gliomas are the most common primary malignancies of the brain, responsible for 2.5% of global tumor-related mortality. They are classified into grades I–IV based on their level of malignancy [1, 2]. Traditionally, histologic diagnosis has been the gold standard for identifying gliomas; however, it is an invasive procedure for patients with intracranial tumors and can be costly, time-consuming, and difficult to reproduce [3]. While medical imaging can offer a preliminary diagnosis of typical gliomas, distinguishing gliomas from other intracranial lesions heavily depends on the radiologist’s expertise. Furthermore, predicting and diagnosing tumors at an early stage remains particularly challenging. This highlights the urgent need to improve non-invasive diagnostic methods for glioma.

Biomarker testing presents a non-invasive approach with high diagnostic potential. Several glioma-associated circulating factors—such as exosomes, cell-free circulating DNA (cfDNA), cell-free circulating RNA (cfRNA), circulating tumor cells, and proteins—could provide a basis for glioma diagnosis [4]. While circulating tumor cells and proteins may yield less timely diagnostic information, cfDNA and cfRNA are prone to degradation in circulation. Exosomes, however, are highly stable, making them more reliable markers for tumor diagnosis [5].

Exosomes, which range from 30 to 100 nm in size, are extracellular vesicles (EVs) involved in the intercellular transport of materials [6]. Their phospholipid bilayer structure protects their contents from degradation in the bloodstream [7, 8]. Exosomes can cross the blood–brain barrier (BBB), circulate in peripheral blood, and transmit cellular signals [9]. Their cargo is highly sensitive to changes in the intracranial microenvironment, which makes exosomes valuable for the early diagnosis of glioma. As a novel circulating biomarker, exosomes enhance the diagnostic accuracy of gliomas compared to traditional screening methods. However, despite their advantages, the diagnostic reliability of exosomes is still limited by biomarker heterogeneity and small sample sizes in studies. To better understand the diagnostic efficacy of exosomes in glioma, we conducted a statistical analysis of previous research to systematically evaluate their performance.

**Figure 1.**
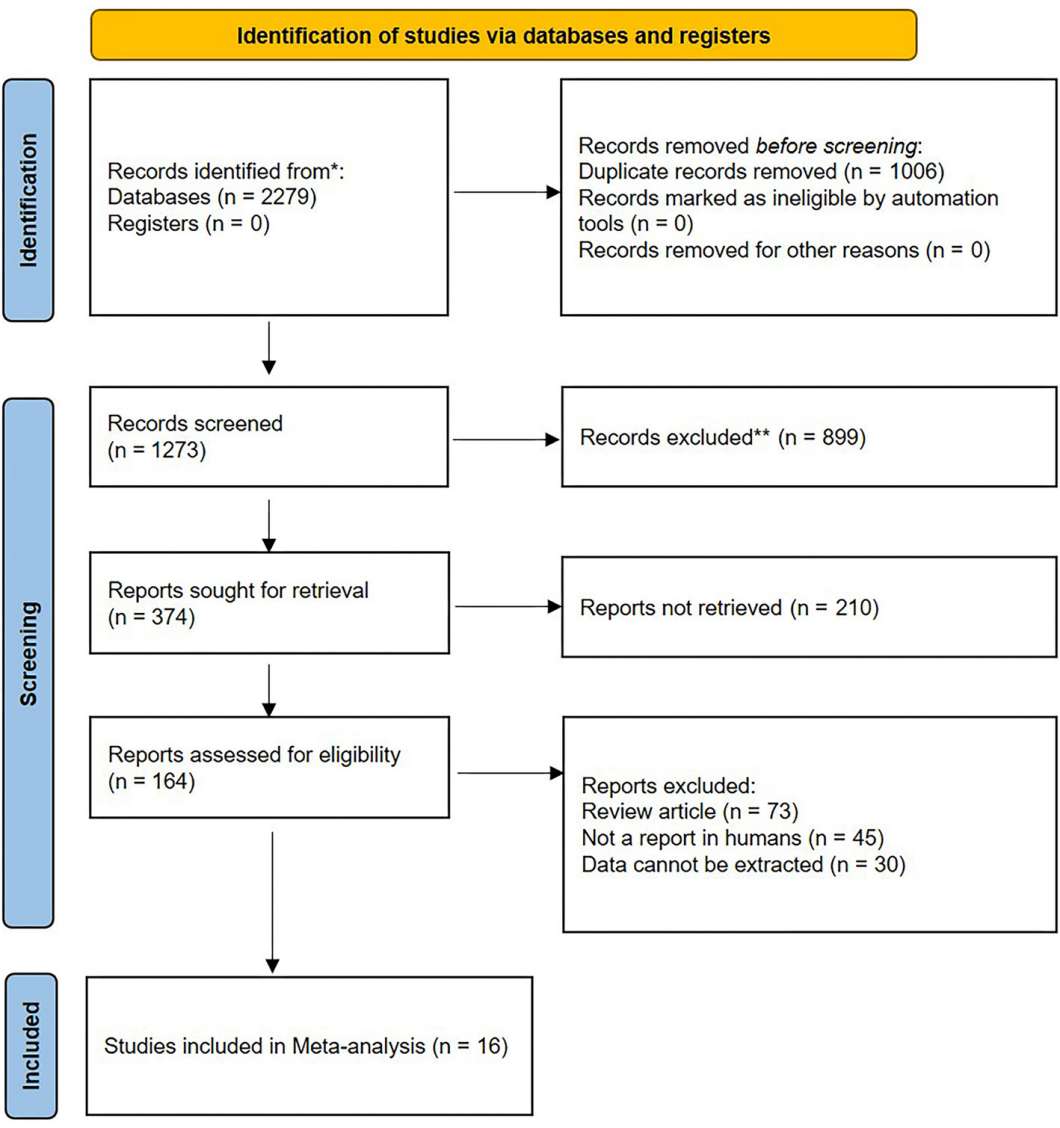
Flowchart of literature search and study selection.

## Materials and methods

### Search strategy

We conducted a search in PubMed and EMBASE using the following keywords: (((extracellular vesicle) OR (extracellular vesicles)) OR (exosome) OR (exosomes) OR (exosomal)) AND (((biomarker) OR (diagnosis) OR (diagnostic) OR (specific) OR (sensitivity) OR (AUC))) AND ((glioma) OR (astrocytoma) OR (glioblastoma)). We also included eligible cited articles in the search scope. This study has been registered in PROSPERO, ID: CRD42024529853.

### Eligibility criteria

Studies were included if they met the following criteria: (1) The study samples were derived from biological fluids of human glioma patients; (2) The article provided sufficient data to directly or indirectly extract true positive (TP), false positive (FP), false negative (FN), and true negative (TN) values.

Exclusion criteria were: (1) Review papers, letters, meta-analyses, case reports, book chapters, and comments; (2) Studies that used the same samples in multiple publications.

### Quality assessment

The quality assessment criteria included: patient selection (continuous and random selection of sample sources), index test (threshold values were determined before evaluation, and results of the test were interpreted without knowledge of the gold standard results), reference standard (the gold standard accurately distinguished diseases, and interpretation was done without knowledge of the index test), and flow and timing (an appropriate interval between the index test and the gold standard). All patients were subject to the same standards, and all cases were included in the analysis.

These indicators were mapped onto the Quality Assessment of Diagnostic Accuracy Studies (QUADAS-2) table to assess publication bias [10]. We also used patient selection, index test, and reference standard criteria to assess applicability concerns. Each assessment index was classified as high risk, low risk, or unclear, with assessments performed independently by two authors (XiangMin Zhang and YanDi Tan). Discrepancies were resolved through discussion with a senior reviewer (JinHua Cai).

**Table 1:** The methods and workflow data from the included studies for glioma diagnosis

**Author**	**Year**	**Country**	**Case/** **control**	**Exosomes source**	**Exosome isolation**	**Biomarker isolation**	**Biomarker analysis**	**TP**	**FP**	**FN**	**TN**
Batool	2022	USA	30/24	Serum	ExoRNeasy	ExoRNeasy	ddPCR	22	1	8	23
Wang	2019	China	23/12	Serum	UC	TRIzol	Real-time PCR	20	2	3	10
Lan	2020	China	91/50	Serum	UC	mirVana	Real-time PCR	75	3	16	50
Lan	2017	China	60/43	Serum	UC	mirVana	Real-time PCR	52	3	8	40
Shao	2019	China	24/24	Serum	Exosome isolation reagent	TRIzol	Real-time PCR	19	2	5	22
Akers	2013	USA	15/14	CSF	UC	mirRCURY kit	Real-time PCR	13	1	2	13
Manterola	2014	USA	50/30	Serum	ExoQuick	TRIzol	Real-time PCR	35	9	15	21
Tan	2018	China	43/40	Serum	Exosome isolation reagent	TRIzol	Real-time PCR	37	5	6	35
Zhong	2019	China	107/80	Serum	ExoQuick	miRNeasy serum/ plasma kit	Reverse transcription PCR	89	15	18	65
Li	2022	China	30/12	Serum	/	mirVana	Real-time PCR	30	0	0	12
Ebrahimkhani	2018	Australia	4/19	Serum	SEC	Exosomal RNA purification mini kit	Deep sequencing	4	0	0	19
Patnam	2022	India	106/20	Serum	Exosome isolation kit	TRIzol	Real-time PCR	76	0	30	20
Manda	2018	India	38/58	Serum	Exosome isolation kit	Total exosome RNA kit	Reverse transcription PCR	31	12	7	46
Shao	2012	USA	24/8	Serum	UC	/	µNMR	22	1	2	7
Figueroa	2017	USA	23/48	CSF	UC	miRNeasy	Real-time PCR	14	1	9	47
Xia	2021	China	100/100	Serum	Exoquick	TRIzol	Real-time PCR	83	13	17	87

CSF: Cerebrospinal fluid; UC: Ultracentrifuged; SEC: Size exclusion chromatography; ddPCR: Droplet digital polymerase chain reaction; PCR: Polymerase chain reaction; µNMR: Miniaturized nuclear magnetic resonance; TP: True positive; FP: False positive; FN: False negative; TN: True negative.

### Data extraction

Two authors (XiangMin Zhang and YanDi Tan) collected relevant information from the included studies, such as the author, year of publication, region, sample size, exosome source, biomarker type, methods of exosome isolation, biomarker isolation, and biomarker analysis. Additionally, TP, FP, FN, and TN data were extracted from 2 × 2 tables or specificity (SPE)/sensitivity (SEN) values reported in the articles. If a training set and validation set were used in the study, only data from the validation set were extracted.

### Ethical statement

Ethical approval and written informed consent were not required for this study in accordance with local/national guidelines.

### Statistical analysis

We performed statistical analyses using Stata 14 and RevMan 5.3. The pooled SEN, SPE, positive diagnostic likelihood ratio (PLR), negative diagnostic likelihood ratio (NLR), and diagnostic odds ratio (DOR) were calculated. The diagnostic value of exosomes in glioma patients was evaluated using the area under the curve (AUC) of the summary receiver operating characteristic (SROC) curve. An AUC of 0.7–0.9 indicated moderate diagnostic accuracy, while an AUC > 0.9 indicated high diagnostic accuracy. All index results were presented as weighted proportions with 95% confidence intervals (CIs).

Cochran’s *Q* test and Higgin’s *I*^2^ statistic were used to assess heterogeneity. *I*^2^ values were used to quantify the magnitude of heterogeneity, with a cut-off of *I*^2^ ═ 50%. If *I*^2^ > 50% indicated significant heterogeneity, a random-effects model was chosen for meta-analysis. If no significant statistical heterogeneity was present, a fixed-effects model was used for the combined analysis. Spearman’s rho and the Moss model b (1) were employed to determine the presence of a threshold effect. A meta-regression analysis was conducted to assess the influence of key study characteristics on outcome indicators, and a subgroup analysis was performed to explore heterogeneity. Publication bias was assessed using Deeks’ funnel plot asymmetry test.

## Results

### Studies and selection

We retrieved 1273 articles from PubMed and EMBASE: 538 from PubMed and 735 from EMBASE. After a preliminary exclusion of 899 articles based on the title, we reviewed the abstracts and excluded 210 more. This left 164 full-text articles, which were evaluated according to inclusion and exclusion criteria. In the end, 16 eligible studies published between 2012 and 2022 were included [11–26]. The document retrieval and screening process is illustrated in Figure 1.

**Table 2:** The exosomal biomarkers for glioma diagnosis in the 16 studies

**Number**	**Author**	**Biomarker**	**Type**
1	Batool	EGFRvIII	Protein
2	Wang	EGFR	Protein
3	Lan	miRNA-210	miRNA
4	Lan	miRNA-301a	miRNA
5	Shao	miRNA-454-3p	miRNA
6	Akers	miRNA-21	miRNA
7	Manterola	sncRNU6-1	Small non-coding-RNA
8	Tan	lncRNA-HOTAIR	Long non-coding-RNA
9	Zhong	miRNA-29b	miRNA
10	Li	hsa:circ_0003828, hsa:circ_0075828, hsa:circ_0002976	Combinations of circular RNA
11	Ebrahimkhani	miR-182-5p, miR-328-3p, miR-339-5p, miR-340-5p, miR-485-3p, miR-486-5p, miR-543	miRNA
12	Patnam	PTEN	Protein
13	Manda	EGFRvIII	Protein
14	Shao	EGFR, EGFRvIII, PDPN, IDH1-R132H	Combinations of protein
15	Figueroa	EGFRvIII	Protein
16	Xia	hsa:circ_0055202, hsa:circ_0074920, hsa:circ_0043722	Combinations of circular RNA

miRNA: MicroRNA; lncRNA: Long non-coding RNA.

### Study characteristics

The extracted data are displayed in Tables 1 and 2. Across the 16 studies, 768 glioma patients in the test groups were diagnosed via histological examination (the gold standard), compared with 582 controls. Exosomes were isolated from serum in 14 studies and from cerebrospinal fluid (CSF) in two. Exosomes were separated using ultracentrifugation (UC), isolation kits, and size exclusion chromatography (SEC). Biomarker analysis of exosomes was performed using quantitative PCR, reverse transcription PCR, droplet digital polymerase chain reaction (ddPCR), deep sequencing, and miniaturized nuclear magnetic resonance (µNMR).

### Quality assessment

**Figure S1.**
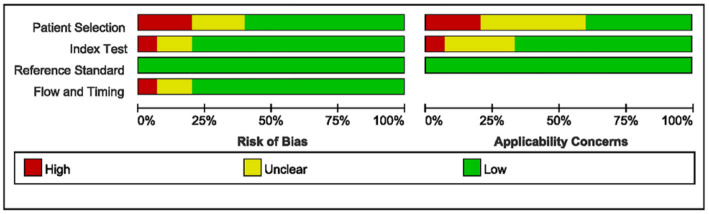
Methodological quality graph.

The QUADAS-2 assessment results are shown in Figure S1. Seven studies were unclear regarding patient selection, index test, and flow/timing, mainly because they did not provide basic patient information (e.g., age and sex) or the methods used for exosome extraction. Some studies also lacked demographic details and the extent of the disease. Additionally, the detection techniques varied, affecting the overall quality. Despite these issues, all studies correctly distinguished the target disease and interpreted the index test results independently of the gold standard results. Overall, the quality assessment based on the QUADAS-2 scale showed that the risk of bias and applicability in these studies was generally good.

### Overall meta-analysis and heterogeneity among studies

**Figure 2.**
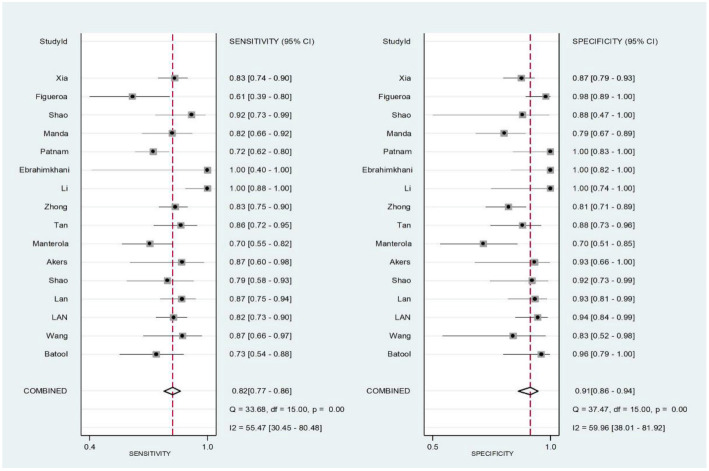
**Pooled estimates of sensitivity and specificity of exosomes for glioma diagnosis.** CI: Confidence interval; I^2^: Inconsistency index.

**Figure 3.**
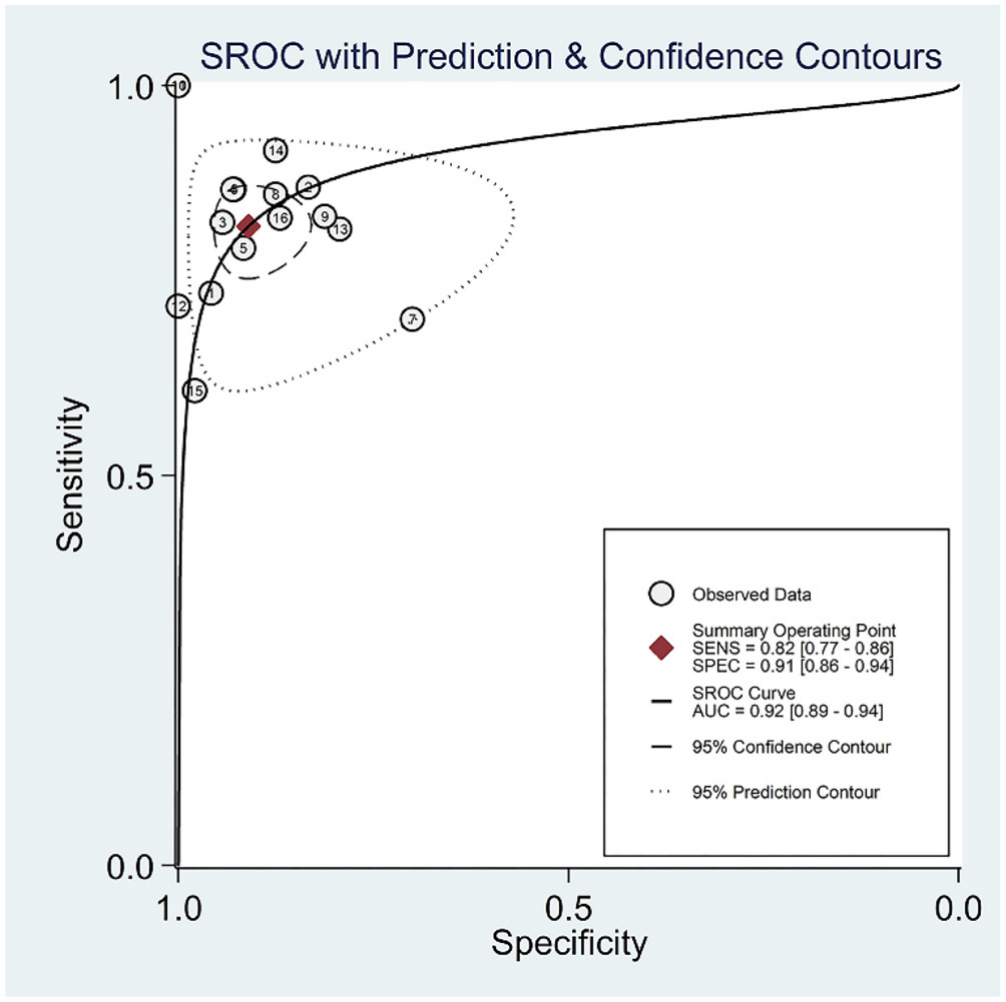
**SROC curve for exosomes derived biomarkers performance in the diagnosis of glioma.** SROC: Summary receiver operating characteristic; AUC: Area under the curve.

**Figure S2.**
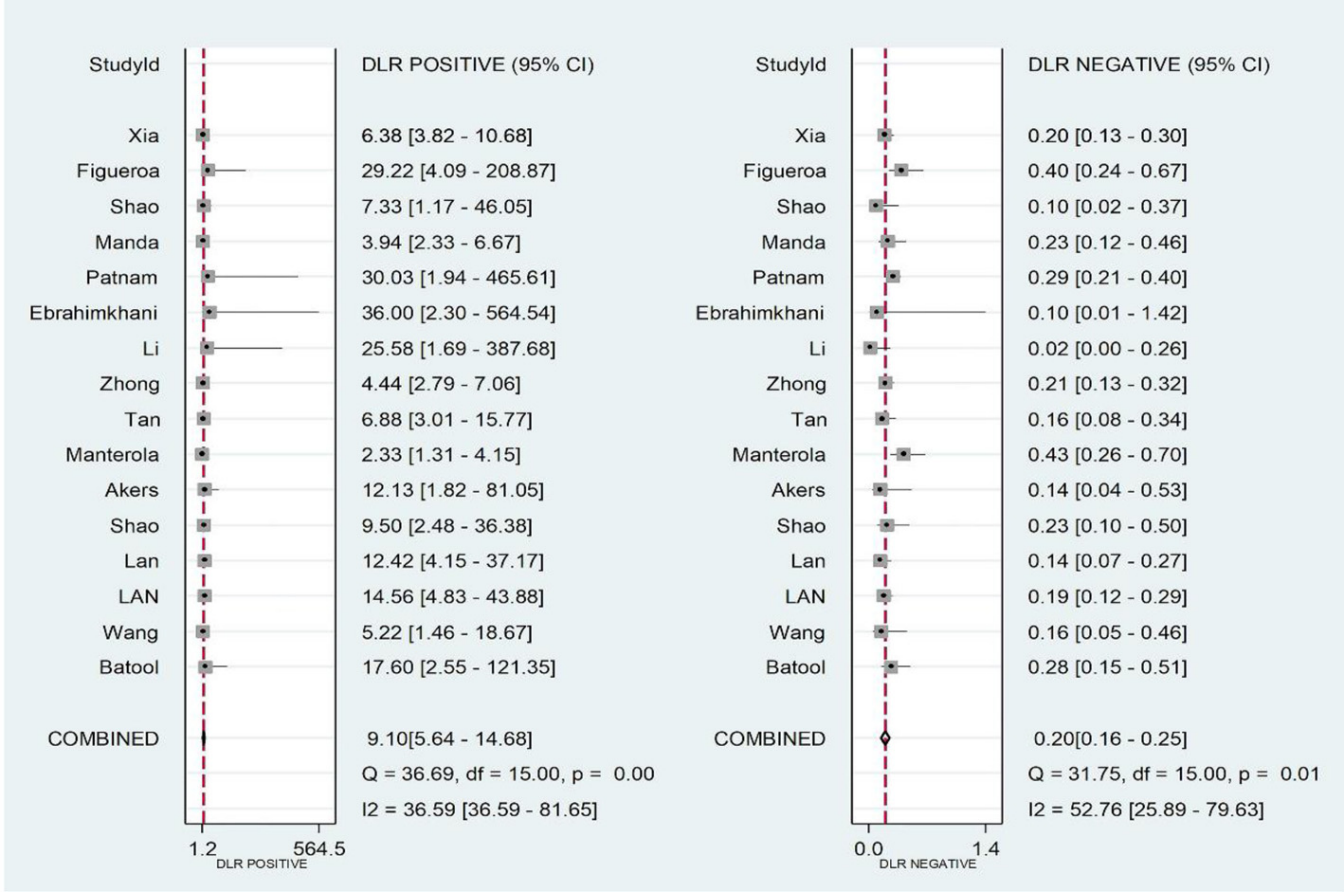
**Coupled forest plots show pooled estimates of positive likelihood ratios and negative likelihood ratios of exosomal biomarkers for glioma diagnosis.** CI: Confidence interval; I^2^: Inconsistency index.

**Figure S3.**
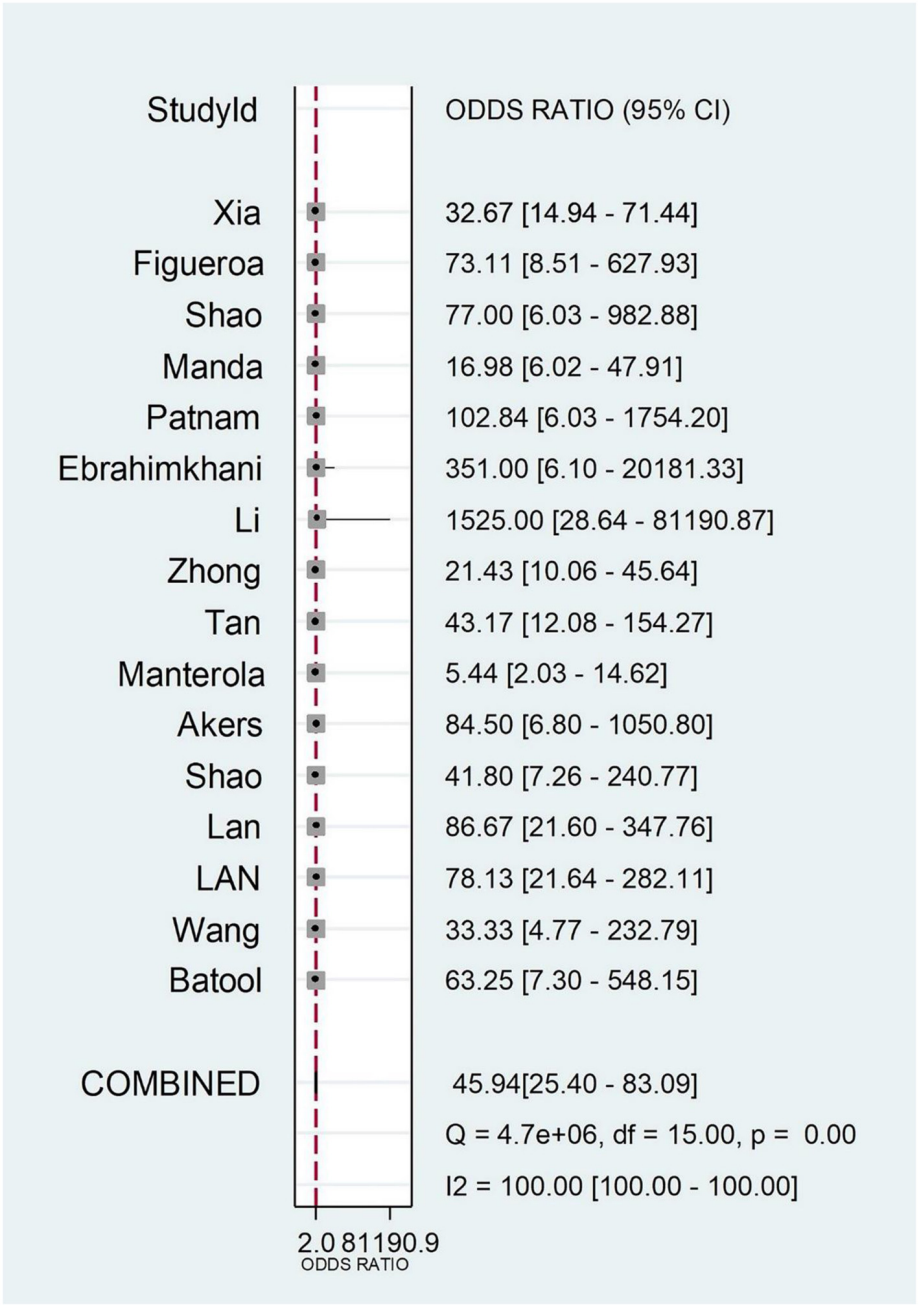
**Forest plot shows pooled estimate of diagnostic odds ratio of exosomal biomarkers for glioma diagnosis.** CI: Confidence interval; I^2^: Inconsistency index.

The pooled SEN, SPE, PLR, NLR, DOR, and AUC for 25 biomarkers across 16 studies were as follows: SEN 82% [95% CI (0.77–0.86)], SPE 91% [95% CI (0.86–0.94)], PLR 9.10 [95% CI (5.64–14.68)], NLR 0.20 [95% CI (0.16–0.25)], DOR 45.94 [95% CI (25.40–83.09)], and AUC 0.92 [95% CI (0.89–0.94)] (Figures 2 and 3 and Figures S2 and S3). Significant heterogeneity was observed for SEN (*I*^2^ ═ 55.47%), SPE (*I*^2^ ═ 59.96%), NLR (*I*^2^ ═ 52.76%), and DOR (*I*^2^ ═ 100%).

**Figure 4.**
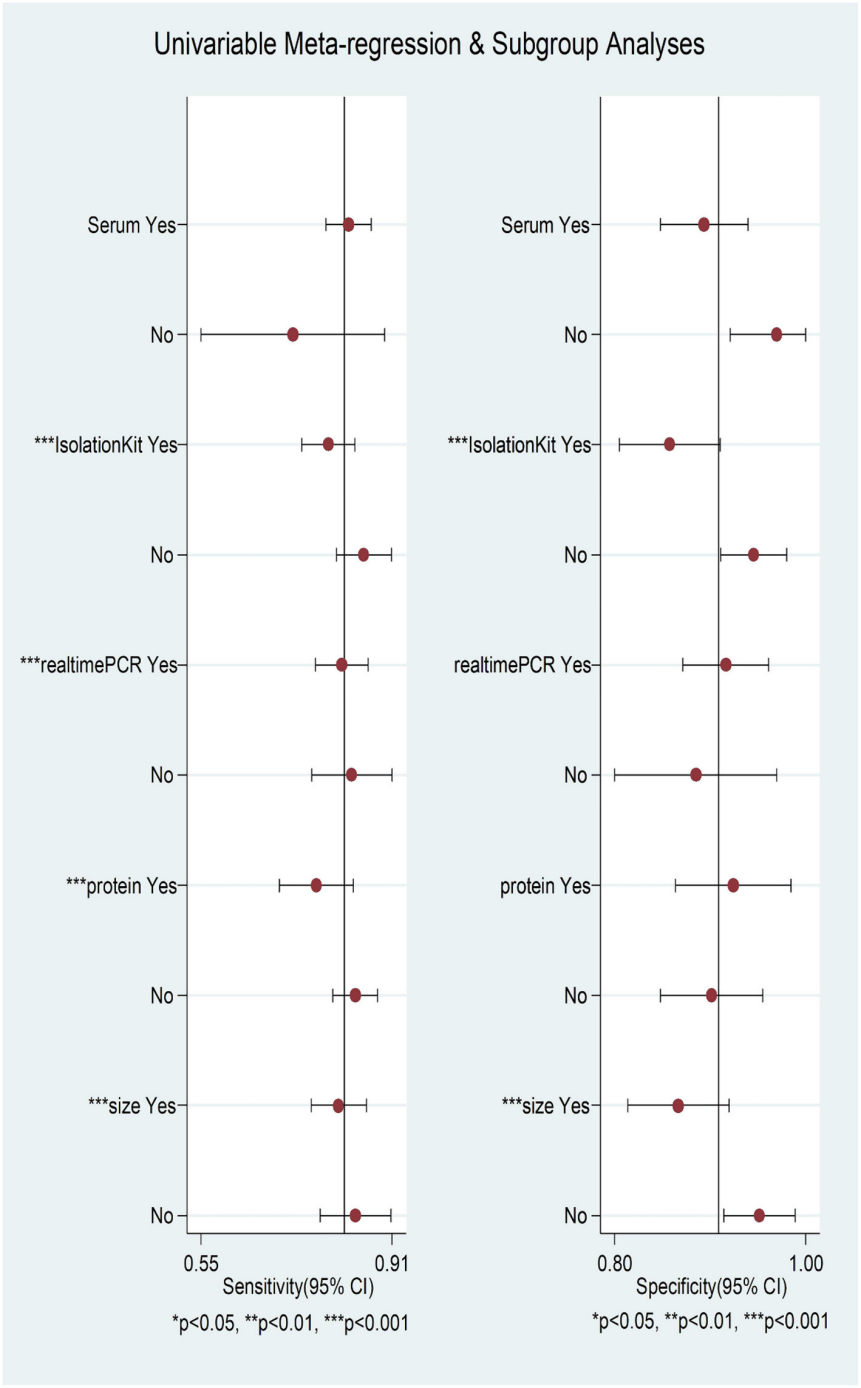
**Coupled forest plots show result of multiple meta-regression and subgroup analysis on pooled sensitivity and specificity**. PCR: Polymerase chain reaction.

**Figure S4.**
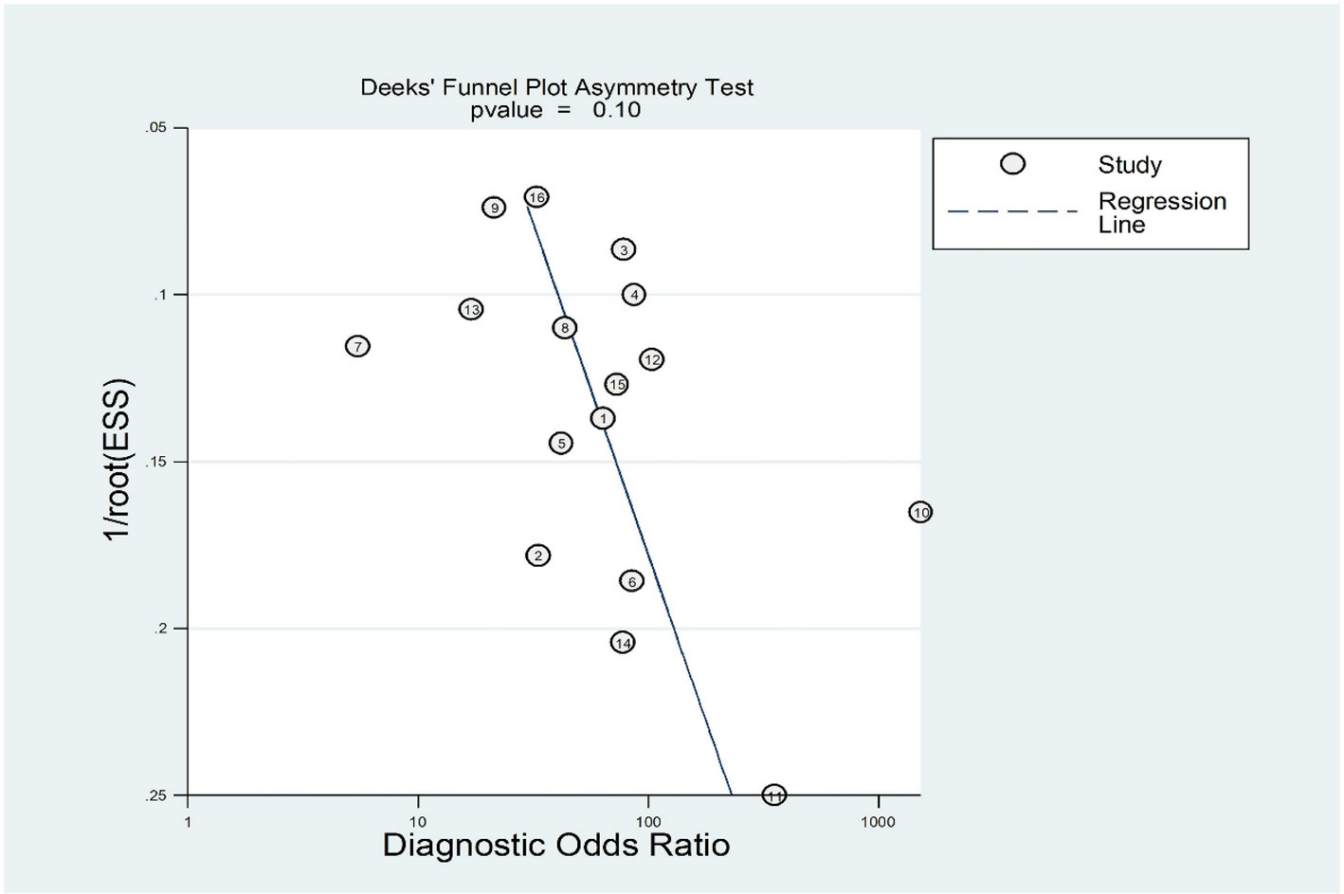
Deeks’ funnel plot asymmetry.

No threshold effect was detected, as indicated by the Spearman correlation coefficient of 0.035 (*P* ═ 0.897) and the *P* value of Moses’ model b (1) ═ 0.0923. We performed a meta-regression analysis based on exosome source (serum or not), exosome isolation method (isolation kit or not), biomarker isolation method (TRIzol or not), biomarker analysis technique (real-time PCR or not), biomarker type (protein or not), and sample size (>30 or not). As shown in Figure 4, the factors influencing pooled SEN heterogeneity were exosome isolation method (*P* < 0.001), biomarker analysis method (*P* < 0.001), biomarker type (*P* < 0.001), and sample size (*P* < 0.001). Similarly, pooled SPE heterogeneity was influenced by biomarker isolation method (*P* < 0.01), exosome isolation method (*P* < 0.001), and sample size (*P* < 0.001). Subgroup analysis results are shown in Table 3. We found that exosomes isolated using UC yielded higher SEN (0.84 vs 0.79), SPE (0.94 vs 0.87), PLR (12.90 vs 5.85), DOR (73.67 vs 23.92), and AUC (0.95 vs 0.86) compared to those isolated with kits. Smaller studies (≤30 samples) demonstrated higher SEN (0.86 vs 0.81), SPE (0.95 vs 0.87), PLR (16.48 vs 6.11), DOR (111.72 vs 27.31), and AUC (0.96 vs 0.89). Exosome marker type and analysis method significantly affected SEN, with microRNA (miRNA) and other groups (small non-coding RNA [sncRNA], long non-coding RNA [lncRNA], and circular RNA [circRNA]) showing higher SEN (0.88 vs 0.84 vs 0.78) compared to protein markers. The “other” group also achieved an AUC as high as 0.95. Interestingly, despite regression analysis indicating that the biomarker analysis method could explain some differences, the SEN was the same (0.82) for both methods, indicating no significant difference. The Deeks funnel plot asymmetry test showed no significant publication bias (*P* ═ 0.1 > 0.05) (Figure S4).

## Discussion

The survival rate for glioma patients is low, particularly for glioblastoma, the most malignant form, which has a median overall survival of only 15 months [27]. Our meta-analysis demonstrated that exosomes exhibit strong diagnostic efficacy, with a pooled SEN of 82% and SPE of 91%. These data indicate that exosomes have an 82% chance of correctly identifying patients with glioma and a 91% chance of correctly identifying healthy individuals. Additionally, the pooled PLR, NLR, DOR, and AUC were 9.10, 0.20, 45.94, and 0.92, respectively. Patients with glioma are 9.1 times more likely to be correctly identified as positive compared to being incorrectly identified as positive in the healthy population. Conversely, patients with glioma are 0.20 times as likely to be incorrectly identified as negative compared to being correctly identified as negative in the healthy population.

Our meta-analysis results surpassed those reported by Jafari et al. [10], likely due to the inclusion of studies conducted after 2020. Advances in the analysis techniques for exosomes since then may have contributed to improved diagnostic efficacy. Additionally, we included studies [20, 21, 24, 26] that used a combination of biomarkers for glioma diagnosis in our statistical analysis. Tumorigenesis and progression involve multiple interacting factors, and combining biomarkers can enhance diagnostic efficiency.

**Table 3:** Pooled estimation of subgroup

**Subgroup**	**Number of studies**	**SEN** **(95% CI)**	**SPE** **(95% CI)**	**PLR** **(95% CI)**	**NLR** **(95% CI)**	**DOR** **(95% CI)**	**AUC** **(95% CI)**
*Sample size*							
>30	8	0.81 (0.76–0.84)	0.87 (0.80–0.92)	6.11 (3.95–9.48)	0.22 (0.18–0.28)	27.31 (15.10–49.39)	0.89 (0.85–0.91)
≤30	8	0.86 (0.74–0.93)	0.95 (0.90–0.97)	16.48 (8.12–33.47)	0.15 (0.08–0.29)	111.72 (39.55–315.59)	0.96 (0.94–0.98)
*Exosomes source*							
Serum	14	0.83 (0.78–0.87)	0.90 (0.84–0.94)	8.67 (5.03–14.94)	0.19 (0.14–0.25)	46.24 (22.03–97.06)	0.92 (0.90–0.94)
CBF	2	--	--	--	--	--	--
*Biomarker type*							
Protein	6	0.78 (0.69–0.85)	0.93 (0.81–0.97)	10.73 (4.03–28.6)	0.24 (0.17–0.33)	45.09 (17.01–119.55)	0.89 (0.86–0.91)
miRNA	6	0.84 (0.79–0.88)	0.92 (0.84–0.96)	10.33 (5.20–20.53)	0.18 (0.13–0.23)	58.74 (25.68–134.38)	0.86 (0.82–0.88)
Other	4	0.88 (0.68–0.96)	0.90 (0.70–0.97)	8.48 (2.26–31.76)	0.14 (0.04–0.45)	62.21 (5.42–713.76)	0.95 (0.92–0.96)
*Exosome isolation*							
Isolation kit	8	0.79 (0.74–0.83)	0.87 (0.79–0.92)	5.85 (3.70–9.23)	0.24 (0.20–0.30)	23.92 (13.80–41.45)	0.86 (0.83–0.89)
UC	6	0.84 (0.75–0.90)	0.94 (0.88–0.97)	12.90 (6.76–24.62)	0.18 (0.12–0.27)	73.67 (36.22–149.84)	0.95 (0.93–0.97)
Other	2	--	--	--	--	--	--
*Biomarker analysis*							
Real-time PCR	11	0.82 (0.76–0.87)	0.92 (0.86–0.95)	9.97 (5.70–17.46)	0.20 (0.14–0.27)	50.48 (24.39–104.49)	0.93 (0.90–0.95)
Other	5	0.82 (0.75–0.88)	0.88 (0.74–0.95)	6.95 (2.99–16.13)	0.20 (0.14–0.29)	34.42 (12.72–93.15)	0.86 (0.83–0.89)

SEN: Sensitivity; SPE: Specificity; PLR: Positive diagnostic likelihood ratio; NLR: Negative diagnostic likelihood ratio; DOR: Diagnostic odds ratio; AUC: Area under the curve; UC: Ultracentrifuged; miRNA: MicroRNA; PCR: Quantitative chain reaction; CI: Confidence interval.

However, our study exhibited significant heterogeneity in SEN (*I*^2^ ═ 55.47%), SPE (*I*^2^ ═ 59.96%), NLR (*I*^2^ ═ 52.76%), and DOR (*I*^2^ ═ 100%). In the absence of publication bias, multivariate meta-regression indicated that factors, such as exosome isolation methods, biomarker analysis techniques, biomarker types, and sample sizes, may contribute to this heterogeneity. Although many studies used isolation kits to extract exosomes, our data suggest that UC-extracted exosomes offer higher diagnostic value. This finding is consistent with Tian et al. [28], who concluded that nFCM analysis highlighted that ExoQuick, TEI, qEV, UF, and exoEasy failed to isolate high-purity EVs from plasma, and UC is the most appropriate isolation method among the ones tested.

Sample size also showed heterogeneity, with studies having a sample size of ≤30 demonstrating higher diagnostic value. This may be due to the tendency of smaller sample studies to amplify effect-size indices. Due to limitations in exosome extraction technologies and sample sources, research on exosomes as biomarkers is often conducted on a small scale. Nevertheless, the exceptional diagnostic value of exosomes for gliomas cannot be ignored.

Our data also indicate that sncRNAs, lncRNAs, circRNAs, and miRNAs are more sensitive biomarkers compared to proteins. This is understandable given that sncRNA, lncRNA, circRNA, and miRNA directly regulate gene expression and are involved in the regulation of the tumor cell cycle. The heterogeneity in biomarker analysis primarily stems from improvements in analytical SEN. As more exosomal biomarkers are identified, they increasingly play a significant role in the diagnosis or prognosis of gliomas, thereby enhancing diagnostic efficacy.

The transcriptional regulation of tumor-specific biomarkers plays a crucial role in tumor progression [29]. These biomarkers can be encapsulated by exosomes, thereby protecting them from degradation in the extracellular environment [30–32]. The use of µNMR [33, 34] and ddPCR [11] has further enhanced the accuracy and reliability of exosomes as biomarkers. Specifically, when µNMR is combined with multiple tumor markers like EGFR, EGFRvIII, PDPN, and IDH1-R132H, the overall diagnostic accuracy has been elevated to over 90% [24]. Facing the limitations of traditional PCR in SENand SPE, the introduction of ddPCR has successfully overcome challenges related to primer dimerization and melting temperature mismatches, solidifying the status of Exo-EGFRvIII as a reliable biomarker [11]. In the field of medical diagnostics, the reliance on single analysis methods based on genetic material is gradually being replaced by more integrated strategies.

Unbiased high-throughput deep sequencing has now made it possible to comprehensively analyze the entire miRNA library in exosomes derived from gliomas. The combined use of miRNAs (miR-182-5p, miR-328-3p, miR-339-5p, miR-340-5p, miR-485-3p, miR-486-5p, and miR-543) has been proven to accurately differentiate among glioma patients, non-tumor controls, and healthy controls [21]. Additionally, circRNAs in exosomes have shown tissue SPE and high diagnostic performance [35]. Integrated circRNAs (hsa:circ_0055202, hsa:circ_0074920, and hsa:circ_0043722) through circRNA microarrays have an AUC of 0.925 [26]. Another study [20] indicates that the diagnostic accuracy of integrated circRNAs based on deep sequencing is as high as 100% (hsa:circ_0075828, hsa:circ_0002976, and hsa:circ_0003828), demonstrating superior diagnostic efficacy compared to previous studies. However, the correlation mechanisms between circRNAs in exosomes and the progression of neurogliomas remain an underexplored area. Further research in this field will not only help deepen our understanding of the functions of circRNAs but also potentially provide more robust theoretical support for their diagnostic applications.

In addition to identifying gliomas, the expression levels of exosomes can also reveal the histological grading of tumors. miR-29b has been proven to effectively distinguish between anaplastic astrocytomas and glioblastomas [19]. About 50% of glioma patients exhibit amplification of the *EGFR* gene, which can be diagnosed and further used to differentiate the malignancy levels of tumors through the detection of Exo-EGFR [36, 37]. Moreover, Exo-miR-210 and Exo-miR-301a increase with the grading of gliomas. Their expression significantly decreases after surgery but rises again during disease recurrence, making them sensitive indicators for monitoring disease dynamics [13, 14]. Notably, patients with low expression of Exo-miR-210 generally have a better prognosis, indicating longer overall survival times. On the other hand, HOTAIR, a lncRNA, regulates tumor progression by participating in chromatin remodeling and promoting the proliferation of gliomas [38]. Exo-HOTAIR shows potential as a biomarker, with an SEN of 86.1% and SPE of 87.5% [18]. As research continues to explore the correlation between Exo-HOTAIR expression levels and glioma subtypes, its potential application could be further expanded.

Biomarkers provide valuable prognostic and predictive factors for glioma patients and may guide physicians in optimizing treatment methods [39]. Additionally, changes in the expression of miRNAs and proteins in exosomes help differentiate between true tumor progression and pseudoprogression. Exo-EGFRvIII not only has a high positive predictive value (93%) and negative predictive value (84%) but also holds promise as a key indicator for assessing the effectiveness of targeted therapies [25]. miR-21, which is highly expressed in many tumor types, is strongly associated with the aggressiveness and malignancy of tumors [40]. The expression of Exo-miR-21 can serve as a biomarker for predicting treatment response and tumor behavior [16]. Therefore, monitoring these biomarkers aids not only in diagnosing tumors but also in tracking biological changes during treatment, thus enabling a more accurate assessment of disease progression.

Exosomes can also serve as prognostic indicators. Pro-oncogenic factors are typically retained in tumor tissues to promote proliferation, while tumor-suppressive factors are released from tumor tissues through exosomes [41]. miR-454-3p and *PTEN*, as tumor suppressor genes, are packaged into exosomes and released from tumors [15, 22]. Low expression of miR-454-3p and *PTEN* in exosomes might suggest that suppressive factors are being retained in tumor tissues, which could serve as an indicator of patients’ overall survival. Previous studies have shown [42, 43] that *PTEN* mutations are associated with glioma staging and overall survival rates.

In addition to serving as biomarkers, exosomes are emerging as a novel tool for tumor therapy. Drug delivery systems based on exosomes demonstrate unique potential in targeted cancer treatments. As naturally occurring nanoscale particles, exosomes can effectively carry therapeutic molecules—including RNAs, DNAs, and proteins—directly to tumor cells, thereby enhancing treatment efficacy while reducing toxicity to normal cells [44]. For example, exosomes can carry doxorubicin to enhance the drug’s accumulation in targeted tumor cells while minimizing toxic side effects to non-target tissues like the heart [45]. By loading regulatory factors, such as siRNAs or miRNAs, exosomes can modulate gene expression within tumor cells, thus inhibiting tumor growth and spread. Exo-miR-122 AMSC has been shown to increase the SEN of hepatocellular carcinoma (HCC) cells to chemotherapy [46].

Immunomodulation is another promising application area for exosomes. Engineered exosomes expressing SIRPα, PD-1, and LILRB1 interact with receptors on tumor cells (CD47, PD-L1, and B2M), promoting the phagocytosis of tumor cells and enhancing antigen presentation by immune cells [47].

## Conclusion

In summary, this study underscores the superior diagnostic efficacy of exosomes as biomarkers for glioma. However, our meta-analysis has several limitations. Meta-regression and subgroup analyses revealed that sample size and biomarker analysis methods contribute to heterogeneity. Additionally, the use of ddPCR and deep sequencing—methods not commonly employed in our experiments—may have introduced further variability. Studies with sample sizes smaller than 30 and inconsistent analytical techniques could potentially exaggerate diagnostic efficacy.

Currently, the lack of standardized methods for extracting and analyzing exosomes is a persistent challenge that contributes to variability in results. This issue is unavoidable at present. Therefore, increasing sample sizes and standardizing the processes of exosome extraction and testing are crucial steps toward advancing research on the clinical application of exosomes. As technological innovations continue, more biomarkers enriched in exosomes will likely be discovered, providing additional empirical evidence to help establish standardized protocols. In the future, exosome-based diagnostics for gliomas may become a low-cost and rapid diagnostic method.

## Supplemental data

## Data Availability

All the data generated during the study are within the manuscript.
